# Pharmacokinetics of a novel endectoparasiticide topical formulation for cats, combining esafoxolaner, eprinomectin and praziquantel

**DOI:** 10.1051/parasite/2021014

**Published:** 2021-04-02

**Authors:** Virginie Jacquot, Prescillia Buellet, Laura Letendre, Wei Tong, Henry Li, Eric Tielemans

**Affiliations:** 1 Boehringer-Ingelheim Animal Health 29 avenue Tony Garnier 69007 Lyon France; 2 Boehringer-Ingelheim Animal Health 631 Route 1 North Brunswick NJ 08902 USA

**Keywords:** Cat, Esafoxolaner, Eprinomectin, Praziquantel, Topical, Pharmacokinetics

## Abstract

Esafoxolaner, a purified enantiomer of afoxolaner with insecticidal and acaricidal properties, is combined with eprinomectin and praziquantel in NexGard^®^ Combo, a novel topical endectoparasiticide formulation for cats. The parasiticide potencies of topical esafoxolaner, eprinomectin and praziquantel, are based on transcutaneous absorption, systemic distribution, and exposure of respective target parasites. For each compound, the pharmacokinetic profile, non-interference, dose linearity/proportionality after one administration, and the accumulation and time to reach a steady state after repeated monthly administrations of the novel formulation, were investigated. After one topical application of NexGard^®^ Combo at the minimum recommended dose, the mean plasma concentration of esafoxolaner immediately reached (and remained at) a level supporting rapid onset and sustained efficacy against ectoparasites for at least 1 month. The mean *C*_max_, *T*_max_, *T*_1/2_, and the topical bioavailability of esafoxolaner were 130 ng/mL, 7.1 days, 21.7 days and 47.2%, respectively, and the plasma profiles of eprinomectin and praziquantel supported their known endoparasiticide properties. No relevant interference between the three compounds was observed. Dose proportionality was demonstrated for the three compounds over a range of 0.5× to 2× the minimum recommended dose. Steady state after repeated monthly administrations was reached by the second dose for praziquantel and by the fifth dose for esafoxolaner and eprinomectin. Accumulation was limited and drug plasma concentrations were maintained within a safe level.

## Abstract (fr)

L’esafoxolaner, un énantiomère purifié d’afoxolaner aux propriétés insecticides et acaricides, est combiné à l’éprinomectine et au praziquantel dans NexGard^®^ Combo, une nouvelle formulation endectoparasiticide topique pour chats. Les pouvoirs parasiticides de l’esafoxolaner topique, de l’éprinomectine et du praziquantel sont basés sur l’absorption transcutanée, la distribution systémique et l’exposition des parasites cibles respectifs. Pour chaque composé, le profil pharmacocinétique, la non-interférence, la linéarité/proportionnalité de dose après une administration, ainsi que l’accumulation et le temps nécessaire pour atteindre un état d’équilibre après des administrations mensuelles répétées de la nouvelle formulation, ont été étudiés. Après une application topique de NexGard^®^ Combo à la dose minimale recommandée, la concentration plasmatique moyenne d’esafoxolaner a immédiatement atteint et est restée à un niveau soutenant une apparition rapide et soutenue de l’efficacité contre les ectoparasites pendant au moins un mois. La *C*_max_ moyenne, la *T*_max_, la *T*_1/2_, et la biodisponibilité topique de l’esafoxolaner était respectivement de 130 ng/mL, 7,1 jours, 21,7 jours et 47,2 %, et les profils plasmatiques de l’éprinomectine et du praziquantel ont confirmé leurs propriétés endoparasiticides connues. Aucune interférence significative entre les trois composés n’a été observée. La proportionnalité de la dose a été démontrée pour les trois composés sur une plage de 0,5 × à 2 × la dose minimale recommandée. L’état d’équilibre après des administrations mensuelles répétées a été atteint par la deuxième dose de praziquantel et par la cinquième dose d’esafoxolaner et d’éprinomectine. L’accumulation était limitée et les concentrations plasmatiques du médicament étaient maintenues à un niveau sûr.

## Introduction

Cats may be affected by multiple parasites, some of them with zoonotic potential [1, 17, 21, 38]. NexGard^®^ Combo, a novel topical endectoparasiticide formulation, combines esafoxolaner, eprinomectin, and praziquantel, and offers a new therapeutic solution to feline veterinary medicine. The parasiticide potency of the three active ingredients in this novel formulation is based on transcutaneous absorption, followed by systemic distribution, and exposure of their respective target parasites. The main objectives of treatment with this novel formulation are to kill existing fleas, ticks and/or ear mites, to prevent new infestations by fleas and/or ticks for at least 1 month, and to kill existing nematodes and cestodes. One topical application should therefore provide an adequate peak plasma concentration for the three active ingredients for rapid onset of activity, and for adequate plasma esafoxolaner levels for sustained efficacy for at least 1 month.

Esafoxolaner, a novel compound, is the purified and active (S)-enantiomer of afoxolaner, a racemic compound from the isoxazoline class. Afoxolaner is commercially available as an oral acaricide and insecticide for dogs, as a single active substance (NexGard^®^) [5] or in combination with milbemycin oxime (NexGard Spectra^®^) [6], a macrocyclic lactone. The acaricidal efficacy of afoxolaner was tested off-label in cats against ear mites and was demonstrated efficacious at 2.5 mg/kg [22]. The advantage of using a purified and active enantiomer is to lower the dose of the active ingredient and consequently lower potential for side effects and chemical and pharmacological interactions (the acaricidal dose of esafoxolaner is 1.44 mg/kg in NexGard^®^ Combo). Esafoxolaner acts as an antagonist at ligand-gated chloride channels, with high specificity for a unique binding site in insect and acarid gamma-aminobutyric acid (GABA)-gated chloride channels. It induces hyper-excitation with uncontrolled activity of the central nervous system and death of insects and acarids [26, 40]. There are no known relevant esafoxolaner binding sites in the mammalian GABA receptors; the selective toxicity of esafoxolaner against insects and acarids versus mammals may be inferred by the differential sensitivity of the arthropods’ GABA receptors versus mammalian counterparts [26, 31]. Following oral administration in dogs with or without milbemycin oxime, the afoxolaner plasma profile shows rapid onset and sustained efficacy against ectoparasites for at least 1 month, with a rapid *T*_max_ and a long half-life [19, 20]. There is also a high correlation between the afoxolaner plasma concentration in dogs and efficacy against fleas and ticks, and the presence of milbemycin oxime does not interfere with afoxolaner efficacy [19]. This was confirmed in clinical studies demonstrating high ectoparasiticide efficacies in dogs for both NexGard^®^ and NexGard Spectra^®^ [9–11, 14, 15, 18, 24, 25, 30].

Eprinomectin is an avermectin, from the macrocyclic lactone class, binding selectively to glutamate-gated chloride ion channels on nerves and muscular cells of several types of invertebrates, including nematodes. Eprinomectin is a well-known compound used in cattle, sheep and goats [27, 33], and cats [12, 29]. Praziquantel is a pyrazino-isoquinoline derivative anthelminthic that acts specifically on cestodes and trematodes, and is a well-known compound in veterinary and human medicine, in oral and topical forms [2, 3, 13, 29, 32, 35, 39].

The pharmacokinetic (PK) profiles of eprinomectin and praziquantel are well-known in Broadline^®^, a topical product for cats combining both active substances with the ectoparasiticide compounds fipronil and (S)-methoprene. Eprinomectin and praziquantel dosages, concentrations and volumes are identical in Broadline^®^ and in NexGard^®^ Combo and have been shown to lack *in vivo* absorption, distribution, metabolism and excretion (ADME) interactions in Broadline^®^ [16].

Nevertheless, since in NexGard^®^ Combo the three active ingredients are in a fixed novel combination, there is a potential for interaction, i.e. an effect on ADME, resulting in a modified PK profile for a specific active substance due to the presence of the other active susbtance(s). It is important to fully understand the relevant PK interactions of the active ingredients in a combination to identify any potential consequence on the efficacy and safety of the product.

Furthermore, as this novel formulation may be administered repeatedly and monthly, it is important to study the level of accumulation of each compound following repeated administration, as there is potential for baseline plasma levels to increase with consequences on efficacy and/or safety.

This manuscript describes the two studies that were carried out to investigate the pharmacokinetic properties of this novel formulation, in which the PK profile of the three compounds, their non-interference, their dose proportionality, and the accumulation effect and time to steady states were studied.

## Materials and methods

### Ethics

The study protocols were reviewed and approved by the Boehringer-Ingelheim Animal Health Inc. Institutional Animal Care and Use Committee (IACUC). Cats were managed and handled similarly and with due regard for their well-being.

### Compliance

Both study designs followed EMEA/CVMP/133/99-FINAL Guidelines for the Conduct of Pharmacokinetic Studies in Target Animal Species, and were conducted in accordance with the Organization for Economic Co-operation and Development (OECD) Principles of Good Laboratory Practice (Revised 1997, issued Jan 1998) ENV/MC/CHEM(98)17.

The two studies were run in the same GLP-certified test facility.

### Animals, husbandry and health

A total of 62 purpose-bred Domestic Short/Long-hair cats sourced from the same licensed breeder were used, 30 cats (15 males and 15 females) weighing 2.2–6.8 kg and aged 9–13 months in Study #1, and 32 (16 males and 16 females) cats weighing 2.7–6.6 kg and aged 6–10 months in Study #2. Each cat was uniquely identified with a microchip and acclimated to the study conditions for at least 10 days before treatment administration. Equal numbers of males and females were randomly allocated to treatment groups based on pre-treatment body weight within sex. Cats were housed indoors, in an environmental-controlled facility, and were group housed by sex and treatment-group, except during the 4 days following treatment when they were individually housed to avoid treatment cross-contamination and to allow individual monitoring. All cats were observed 2 and 4 h after treatment application and once daily throughout the study, for the monitoring of health abnormalities and adverse reactions.

### Treatments and blood samplings

All topical treatments were applied on one spot directly on the skin, after parting the hair, in the midline of the neck between the base of the skull and the shoulder blades. The intra-venous injection was given in the cephalic vein.

All blood samples were collected into lithium heparin tubes and processed into plasma by centrifugation. For animal welfare purposes, the individual blood sample volumes were limited to avoid exceeding cumulative blood withdrawal of approximately 15% of circulating volume in 4 weeks.

## Study designs

Designs of Studies #1 and #2 are summarized in Table 1.

**Table 1:** Study designs.

Study/Group	*n*^6^	Dosage (mL/kg)	Treatment Day(s)	Blood collection days (minutes, hours after treatment)
Study #1 – Pharmacokinetics and non-interference				
NexGard^®^ Combo – Topical^1^	8	0.12	0	0 (4 h, 8 h), 1 (24 h, 32 h), 2, 4, 7, 14, 21, 29, 42, 56, 70
Esafoxolaner – Topical^2^	8	0.12	0	0 (4 h, 8 h), 1 (24 h, 32 h), 2, 4, 7, 14, 21, 29, 42, 56, 70
Broadline^®^ – Topical^3^	8	0.12	0	0 (4 h, 8 h), 1(24 h, 32 h), 2, 4, 7, 14, 21, 29, 42, 56, 70
Esafoxolaner – Intravenous^2^	6	0.12	0	0 (15 min, 30 min, 2 h, 4 h, 8 h), 1 (24 h, 32 h), 2, 4, 7, 14, 21, 29, 42, 56, 70
Study #2 – Dose proportionality and multiple-dose kinetics				
NexGard^®^ Combo – Topical– 0.5×^4^	8	0.06	0	0 (2 h, 4 h, 8 h), 1 (24 h, 32 h), 2, 4, 7, 14, 21, 28, 42, 56,70, 84 and 91
NexGard^®^ Combo – Topical– 1× 1	8	0.12	0	0 (2 h, 4 h, 8 h), 1 (24 h, 32 h), 2, 4, 7, 14, 21, 28, 42, 56,70, 84 and 91
NexGard^®^ Combo – Topical – 2× 5	8	0.24	0	0 (2 h, 4 h, 8 h), 1 (24 h, 32 h), 2, 4, 7, 14, 21, 28, 42, 56,70, 84 and 91
NexGard^®^ Combo – Topical– 1×^1^	8	0.12	0, 28, 56, 84, 112	0 (2 h, 4 h, 8 h), 1 (24 h, 32 h), 2, 4, 7, 14, 21, 28*, 56*, 84*, 112 (0 h*, 2 h, 4 h, 8 h), 113 (24 h, 32 h), 114 (48 h), 116, 119, 126, 133, 140, 154 and 168

* Blood sampling before treatment.

1 Esafoxolaner 1.44 mg/kg, praziquantel 10.0 mg/kg, eprinomectin 0.5 mg/kg.

2 Esafoxolaner alone 1.44 mg/kg.

3 Fipronil 10.0 mg/kg, (S)-methoprene 12.0 mg/kg, praziquantel 10.0 mg/kg, eprinomectin 0.5 mg/kg.

4 Esafoxolaner 0.72 mg/kg, praziquantel 5.0 mg/kg, eprinomectin 0.25 mg/kg.

5 Esafoxolaner 2.88 mg/kg, praziquantel 20.0 mg/kg, eprinomectin 1.0 mg/kg.

6 *n* = number of cats per group (equal number of males and females).

### Study #1

This study was designed to evaluate the main pharmacokinetic parameters (*C*_max_, *T*_max_, *C*_last_, *T*_last_, half-life, AUC, bioavailability) of the three compounds administered topically once in the novel formulation at the recommended minimum dose, and the non-interference of the three compounds between each other. A comparison with Broadline^®^, a topical endectoparasiticide product for cats in which eprinomectin and praziquantel are present in identical concentrations, was also used for analysis of non-interference in relation to the two endoparasiticide compounds.

Group 1 was treated topically with NexGard^®^ Combo at the minimum recommended dose for analysis of the plasma profile of esafoxolaner, eprinomectin and praziquantel. Group 2 was treated topically with esafoxolaner alone, formulated with identical solvents/excipients as those of the novel formulation, for non-interference evaluation of esafoxolaner. Group 3 was treated with Broadline^®^ for comparison of plasma levels of eprinomectin and praziquantel, and partial bridging of pharmacokinetic data from Broadline^®^ for non-interference evaluation of eprinomectin and praziquantel in the novel formulation. Group 4 was treated intravenously with esafoxolaner for bioavailability calculation of topical esafoxolaner in the novel formulation.

Blood samples were collected prior to treatment and over a period of 70 days after treatment as detailed in Table 1.

### Study #2

This study was designed to evaluate the dose proportionality/linearity of esafoxolaner, eprinomectin and praziquantel, after a single topical administration of the novel formulation at different doses, and to evaluate accumulation and time to steady state of the three active ingredients following repeated topical administrations.

Groups 1, 2 and 3, the dose proportionality/linearity groups, were treated once topically with 0.5, 1 or 2 times the minimum recommended dose volume of NexGard^®^ Combo, respectively. Blood samples were collected prior to treatment and over a period of 91 days after the treatment, as detailed in Table 1.

Group 4, the accumulation and time to steady state group, was treated five times at 28-day intervals, topically with NexGard^®^ Combo at the minimum recommended dose. In this group, blood samples were collected prior to each treatment, during the 4 weeks following each treatment, and for 8 weeks after the last treatment, as detailed in Table 1.

## Plasma analysis of esafoxolaner, eprinomectin and praziquantel

Esafoxolaner, eprinomectin and praziquantel were quantitatively analyzed using liquid chromatography coupled to tandem mass spectrometry (LC-MS/MS). The cat plasma samples and internal standards were subjected to semi-automated solid phase extraction in a 96-well plate format followed by reversed-phase HPLC with gradient elution. The extracted analytes were quantified by an AB Sciex API 5000 mass spectrometer system using an electrospray interface. Drugs and internal standards were detected in positive ionization mode using multiple reaction monitoring of the precursor to product ions transition of m/z 626.2 → 470.3, 313.3 → 203.2, and 914.5 → 186.2 for esafoxolaner, praziquantel, and eprinomectin B1a, respectively. Chromatograms were integrated by the Analyst^®^ 1.6.2 software and peak areas were determined.

The validated lower and upper limits of quantitation were 0.2 and 100 ng/mL, respectively. Sample concentrations were determined based on a fortified matrix calibration curve ranging from 0.2 to 100 ng/mL for each analyte with quality control samples, control plasma samples, and acceptance criteria according to EMA and FDA guidelines [4, 7]. Repeatability (precision), accuracy, assay specificity, dilution, recovery and matrix effect, carryover, stability in plasma and all solutions was established.

### Pharmacokinetics analysis

Pharmacokinetic (PK) parameters were calculated from individual plasma concentrations using the non-compartmental analysis function of WinNonlin^®^ software (Phoenix 64 Build, version 6.3). The non-compartmental approach was consistent with the extravascular (topical) or IV bolus route of administration.

The peak drug plasma concentration *C*_max_ and the time from dosing to the maximum concentration values *T*_max_ were obtained directly from plasma concentration versus time data for each cat and then averaged for each topical treatment group. The terminal elimination phase was determined using at least the final three observed concentrations and not including *C*_max_. The first order rate constant, *λ*_z_, associated with the terminal log-linear portion of the curve was estimated via linear regression of the log plasma concentration versus time curve. The terminal plasma half-life using ln (2)/*λ*_z_ was calculated for each animal, and then averaged for each treatment group. The area under the concentration versus time curve (AUC) from the time of dosing to the time to the last quantifiable concentration (AUC_0−Tlast_) was calculated using the linear up, log down trapezoidal method, and extrapolated to infinity (AUC_0−inf_) using the formula AUC_0−Tlast_ + *C*_last_/*λ*_z_.

Bioavailability (%F) of esafoxolaner in the NexGard Combo treated group was determined as the mean dose-normalized AUC_0−inf_ (topical)/AUC_0−inf_ (IV) for the active substance. Bioavailability of eprinomectin and praziquantel was determined in other studies. The systemic clearance of esafoxolaner in plasma (Cls) defined as dose/AUC_0−inf_, and the volume of distribution at steady state (*V*_ss_), which is AUMC_0−inf_/AUC_0−inf_ × Cls, was calculated from WinNonlin^®^ for the IV esafoxolaner group only. Due to the long terminal plasma half-life of esafoxolaner, each cat was not used as its own control and bioavailability was calculated on the average. In order to determine and compare the PK parameters of eprinomectin and praziquantel when administered topically with esafoxolaner in NexGard^®^ Combo or within Broadline^®^, statistical evaluations of the PK parameters for different formulations were performed using a paired student’s *t*-test and a *p*-value < 0.05 was used as the criterion for difference.

Dose proportionality for the three active ingredients was assessed by calculating the linear relationship between AUC or *C*_max_ and the dose using the simple linear regression model.

Accumulation ratios were calculated using *C*_max_ and AUC for the three active ingredients after the 1st and 5th dose following five topical doses of the novel formulation. Furthermore, steady state was assessed using paired student’s *t*-test on esafoxolaner, eprinomectin and praziquantel concentrations determined immediately prior to each dose of the novel formulation (*p*-values result from a comparison of two consecutive doses).

## Results

### Pharmacokinetics

Pharmacokinetic parameters of esafoxolaner administered topically in the novel formulation, topically alone, intravenously alone, and of eprinomectin and praziquantel administered topically in the novel formulation and in Broadline^®^ are presented in Table 2.

**Table 2:** Summary of the pharamacokinetic parameters (mean ± *SD*) of esafoxolaner, eprinomectin and praziquantel in NexGard^®^ Combo following a single topical application at the minimum recommended dose, of eprinomectin and praziquantel in Broadline^®^, and of esafoxolaner alone administered topically or intravenously.

Analyte	Group^1^	*T*_1/2_	*T*_max_	*C*_max_	AUC_0−Tlast_	AUC_0−inf_	Cl	*V*_ss_
		(day)	(day)	(ng/mL)	(day × ng/mL)	(day × ng/mL)	(mL/day/kg)	(mL/kg)
Esafoxolaner –Topical	1	21.7 ± 2.8	7.13 ± 3.1	130 ± 36	4411 ± 1525	4972 ± 1711	NA	NA
	2	20.8 ± 6.4	3.75 ± 3.1	218 ± 68	6368 ± 1665	7181 ± 2250	NA	NA
	*p*-value^2^	0.713	0.019	0.0305	0.0792	0.0923	NA	NA
Eprinomectin –Topical	1	5.42 ± 2.7	1.46 ± 0.47	23.6 ± 11	156 ± 94	159 ± 94	NA	NA
	3	5.63 ± 1.6	1.71 ± 0.97	27.1 ± 16	175 ± 67	179 ± 69	NA	NA
	*p*-value^2^	0.806	0.476	0.6	0.656	0.642	NA	NA
Praziquantel – Topical	1	4.30 ± 1.9	0.292 ± 0.08	107 ± 59	123 ± 25	132 ± 23	NA	NA
	3	3.50 ± 2.2	0.313 ± 0.06	118 ± 88	185 ± 93	173 ± 89	NA	NA
	*p*-values^2^	0.462	0.351	0.797	0.0897	0.187	NA	NA
Esafoxolaner –Intravenous	4	21.0 ± 4.2	NA	NA	9586 ± 1985	10,542 ± 2029	141 ± 29.5	4016 ± 1190
	*p*-value^2^ vs. 1	0.657	NA	NA	NA	NA	NA	NA
	*p*-value^2^ vs. 2	0.484	NA	NA	NA	NA	NA	NA

*T*_1/2_ = plasma half-life; *T*_max_ = time from dosing to the maximum concentration; *C*_max_ = peak drug plasma concentration; AUC = area under the concentration versus time curve: 0−Tlast = from the time of dosing to the time to the last quantifiable concentration, 0−inf = from the time of dosing to infinity (by extrapolation); Cl = systemic clearance; *V*_ss_ = volume of distribution at steady-state.

1 Group 1: NexGard Combo – Topical; esafoxolaner 1.44 mg/kg, eprinomectin 0.5 mg/kg, praziquantel 10.0 mg/kg. Group 2: Esafoxolaner alone – Topical; esafoxolaner 1.44 mg/kg. Group 3: Broadline^®^ – Topical; fipronil 10.0 mg/kg, (S)-methoprene 12.0 mg/kg, eprinomectin 0.5 mg/kg, praziquantel 10.0 mg/kg. Group 4: Esafoxolaner – IV; esafoxolaner 1.44 mg/kg.

2 Paired student’s *t*-test.

#### Esafoxolaner

**Figure 1:**
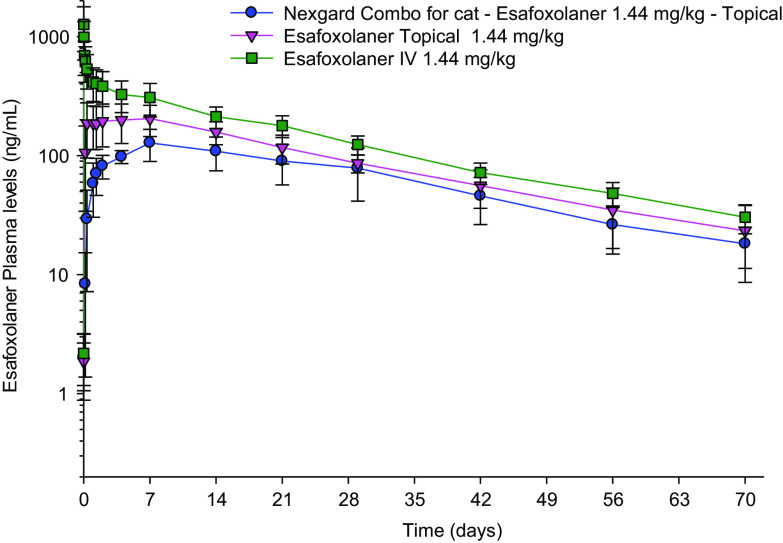
Esafoxolaner average concentration–time curves, when administered in NexGard^®^ Combo, or alone in a topical or in an intravenous formulation.

The esafoxolaner average concentration–time curves are plotted in Figure 1.

After a single topical application of the combined formulation at the minimum recommended dose, esafoxolaner (applied at 1.44 mg/kg) increased up to a mean *C*_max_ of 130 ± 36 ng/mL, reached at a mean *T*_max_ of 7.13 ± 3.1 days (the mean esafoxolaner concentration 24 h after treatment was 58.6 ± 28.2 ng/mL). Concentrations then declined steadily and were quantifiable for at least 10 weeks with mean concentration of 18.2 ± 6.9 ng/mL 70 days after dosing. Mean AUC_0−Tlast_ was 4411 ± 1525 day × ng/mL, and the individual plasma half-lives ranged from 19.4 to 27.8 days (mean = 21.7 ± 2.8 days). The full pharmacokinetic curve was captured and less than 20% of the AUC was extrapolated for all animals.

The comparison of esafoxolaner administered topically in the novel formulation (combined with eprinomectin and praziquantel), and administered topically alone, showed no significant difference for *T*_1/2_, *C*_last_ and AUCs (AUC_0−Tlast_ and AUC_0−inf_) (*p* ≤ 0.08), and a significant difference for *C*_max_ and *T*_max_ (*p* = 0.031 and *p* = 0.019, respectively).

Intravenous administration of esafoxolaner at 1.44 mg/kg revealed a mean half-life of 21.0 ± 4.2 days (ranging from 14.8 to 26.3 days), similar to values obtained after topical application in the combined formulation or alone (*p* = 0.657 and 0.484, respectively), indicating that the topical terminal plasma half-life was not absorption-limited. The clearance and volume of distribution were 141 ± 29.5 mL/day/kg and 4016 ± 1190 mL/kg, respectively. Topical esafoxolaner in the novel formulation was absorbed with an absolute bioavailability of 47.2%.

#### Praziquantel

**Figure 2:**
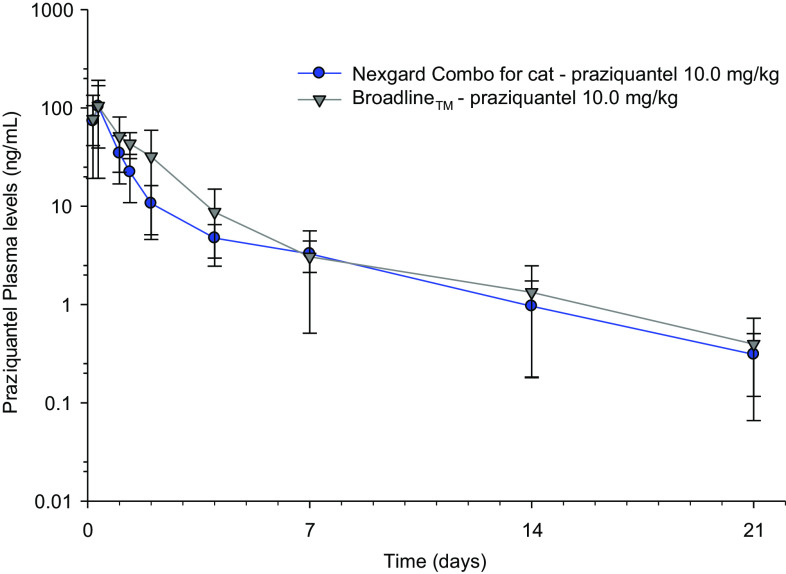
Praziquantel average concentration–time curves, when administered in NexGard^®^ Combo, or in Broadline^®^.

The praziquantel average concentration–time curves are plotted in Figure 2.

After a single topical administration of NexGard^®^ Combo, praziquantel concentrations peaked quickly, indicating rapid absorption. The maximum concentrations were reached in 4 to 8 h; the mean *C*_max_ was 107 ± 59 ng/mL. Concentrations then declined steadily with the last quantifiable plasma concentrations reached between 7 and 42 days following treatment. The mean half-life was 4.3 ± 1.9 days and the mean AUC_0−Tlast_ was 123 ± 25 day × ng/mL. The full pharmacokinetic curve was captured and less than 20% of the AUC was extrapolated for all animals. When administered in Broadline^®^, all PK parameters of praziquantel were not significantly different to those of the novel formulation. The bioavailability of praziquantel had been determined in the Broadline formulation to be 45% [16].

#### Eprinomectin

**Figure 3:**
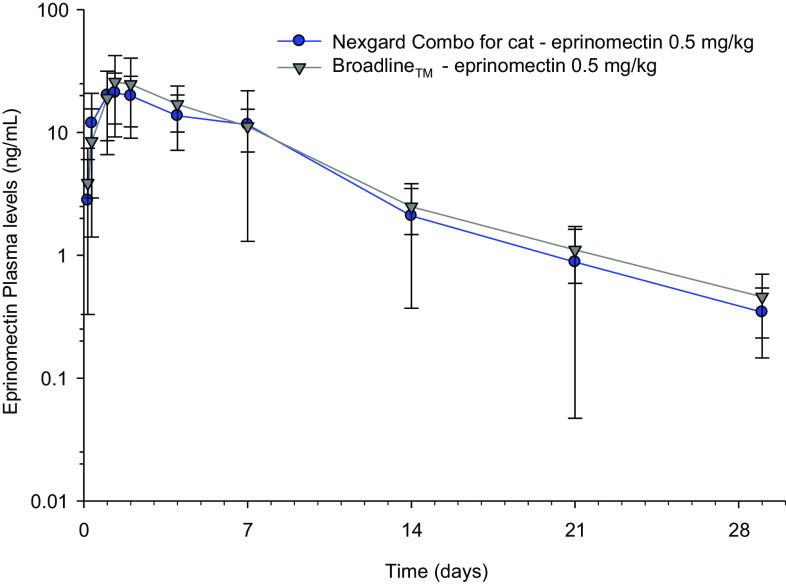
Eprinomectin average concentration–time curves, when administered in NexGard^®^ Combo, or in Broadline^®^.

The eprinomectin average concentration–time curves are plotted in Figure 3.

After a single topical administration of NexGard^®^ Combo, for eprinomectin, a mean *C*_max_ of 23.6 ± 11.0 ng/mL was achieved at a mean *T*_max_ of 35 h, and was followed by a gradual decrease in concentrations below the limit of quantitation after, on average, 28 days. The mean half-life was 5.4 ± 2.7 days and the mean AUC_0−Tlast_ was 156 ± 94 day × ng/mL. The full pharmacokinetic curve was captured and less than 20% of the AUC was extrapolated for all animals. When administered in Broadline^®^, all PK parameters of eprinomectin were not significantly different to those of the novel formulation. The bioavailability of eprinomectin had been determined in the Broadline formulation to be 31% [16].

#### Dose proportionality/linearity of the novel formulation

**Figure 4:**
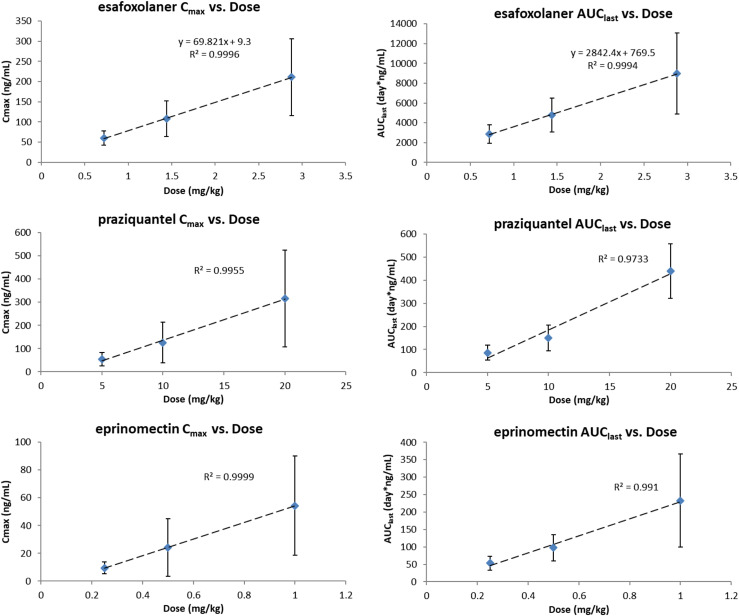
Plots of mean C_max_ values and AUC_last_ values as a function of increasing dose for esafoxolaner, praziquantel and eprinomectin.

Plots of mean *C*_max_ and AUC_last_ values as a function of increasing dose are shown in Figure 4.

After a single topical administration of three increasing doses of the combined formulation (0.5×, 1× and 2× the intended minimum label dose), C_max_ and AUC_0-Tlast_ of the three active substances increased approximately proportionally with the dose, as illustrated in Table 3.

**Table 3:** Mean *C*_max_ and AUC for esafoxolaner, eprinomectin and praziquantel following a topical single administration 0.5, 1 or 2 times the intended minimum dose of the novel formulation.

Dose Level	Dose (mg/kg)	*C*_max_ (ng/mL)	AUC_0−Tlast_ (day × ng/mL)	*C*_max_ Ratio	AUC_0−Tlast_ Ratio
Esafoxolaner					
0.5×	0.72	60.8 ± 17.7	2873 ± 931	0.6	0.6
1×	1.44	108 ± 43.7	4777 ± 1714	–	–
2×	2.88	211 ± 94.8	8984 ± 4071	2.0	1.9
Praziquantel					
0.5×	5	54.7 ± 28.8	86.5 ± 33.0	0.5	0.6
1×	10	126 ± 87.2	150 ± 56.1	–	–
2×	20	317 ± 209	440 ± 118	2.5	2.9
Eprinomectin					
0.5×	0.25	9.44 ± 4.26	53.6 ± 19.9	0.4	0.5
1×	0.5	24.1 ± 20.8	97.8 ± 37.4	–	–
2×	1	54.3 ± 35.8	233 ± 133	2.3	2.4

*C*_max_ = peak drug plasma concentration; AUC_0−Tlast_ = area under the concentration versus time curve, from the time of dosing to the time to the last quantifiable concentration.

For esafoxolaner, praziquantel and eprinomectin, the 0.5× and the 2× groups, compared to the 1× group, had average *C*_max_ and AUC ratios ranging from 0.4 to 0.6 and 1.9 to 2.9, respectively. These results indicate that the PK parameters of esafoxolaner, praziquantel and eprinomectin are dose-proportional over the dosing ranges of 0.72–2.88 mg/kg, 5–20 mg/kg, and 0.25–1.0 mg/kg, i.e. 0.5× to 2× the minimum dose, respectively, following a single topical dose of NexGard^®^ Combo.

#### Multiple dose kinetics of the novel formulation

**Figure 5:**
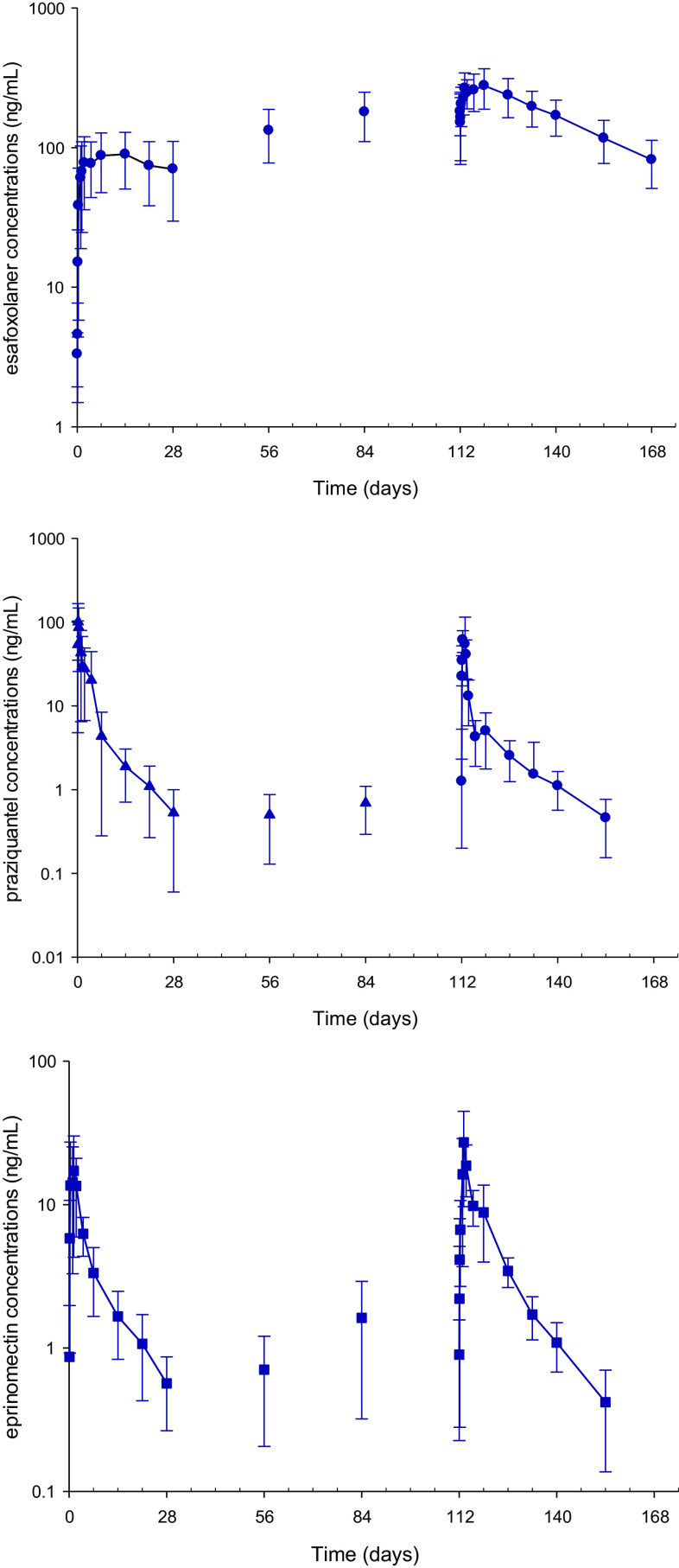
Mean plasma concentrations of esafoxolaner, praziquantel and eprinomectin after five topical administrations of NexGard^®^ Combo at 4-week intervals.

Mean plasma concentrations of esafoxolaner, praziquantel and eprinomectin after five topical administrations of NexGard^®^ Combo at 4-week intervals are presented in Figure 5.

The AUC accumulation factor for esafoxolaner was 3.2 and the steady state was reached by the fifth 4-weekly dose. The AUC accumulation factor for eprinomectin was 2.1 and the steady state was reached by the fifth 4-weekly dose. No apparent accumulation (accumulation factor of ~1) was observed for praziquantel after five doses at 4-week intervals and the steady state was reached by the second dose.

### Tolerance

None of the 62 cats included in both studies experienced any adverse reactions related to any of the treatments.

## Discussion

The PK profile of esafoxolaner in NexGard^®^ Combo was characterized by a long persistence, i.e. a quantifiable concentration lasting more than 13 weeks after a single administration at minimum dose due to high plasma protein binding, a high volume of distribution (*V*_ss_ = 4.0 L/kg), and a very low intrinsic clearance resulting in a long terminal half-life of 21 days. The PK profile of esafoxolaner in the combined formulation was in accordance with ectoparasiticide objectives of rapid onset and sustained efficacy of at least one month. The EC_90_ of esafoxolaner is 19.1 ± 2.1 ng/mL for *C. felis* and 43.1 ng/mL for ticks (*Ixodes scapularis*) [internal Sponsor’s data], and these efficacious plasma levels were maintained from 24 h until 6–8 weeks after a single application. This was confirmed by efficacy data obtained with NexGard^®^ Combo against fleas [34, 37] and ticks [28, 36] in cats.

Eprinomectin and praziquantel plasma profiles following a topical application of the combined formulation have systemic endoparasiticide concentrations needed to kill nematodes and cestodes. Both compounds also have appropriate PK parameters (AUC, *C*_max_ and *T*_max_), that are identical to those of Broadline^®^, a product with proven efficacy against nematodes and cestodes [12, 29, 35]. This was confirmed by efficacy data obtained with NexGard^®^ Combo against both nematodes and cestodes [13] in cats.

Esafoxolaner did not interfere with the ADME properties of eprinomectin and praziquantel. The AUC, half-life and *C*_last_, the most important parameters for sustained preventive flea and tick efficacy were unchanged when the active ingredient was given as a fixed combination or alone. However, the higher *C*_max_ achieved in less time (shorter *T*_max_) for esafoxolaner administered alone, indicated possible slower absorption of esafoxolaner when administered in the novel formulation (in combination with eprinomectin and praziquantel). Nevertheless, this has no impact on the onset of curative efficacy of NexGard^®^ Combo, as consistently confirmed by 24-hour and 48-hour high levels of efficacy after treatment against fleas and ticks, respectively [28, 34, 36]. Eprinomectin and praziquantel had similar plasma profiles after administration in Broadline^®^ or in NexGard^®^ Combo, which allows the bridging of non-interference conclusions of these two compounds from the Broadline^®^ studies [16].

Evaluations of plasma concentrations of the three compounds following administration of the novel formulation at half, equal, and double the minimum recommended dose demonstrated dose proportionality and thus linearity of ADME properties.

Evaluations of plasma concentrations of the three compounds following repeated topical treatments at 4-week intervals with the novel formulation demonstrated that a steady state was reached after five treatments for esafoxolaner and eprinomectin, and after two treatments for praziquantel. At steady state, the accumulation factor was 3.2 for esafoxolaner, 2.1 for eprinomectin and 1.0 (i.e. no accumulation) for praziquantel. Feline tolerance to the novel formulation was investigated in several target animal safety studies, namely two margin of safety studies [8]. In the first (pilot) study, two groups of cats were treated with the novel formulation at 3× and 5× multiples of the maximum exposure dose, four times at 2-week intervals, and one group was treated with a topical formulation of only esafoxolaner, twice at a 28-day interval, providing 23× the maximum exposure dose of esafoxolaner in NexGard^®^ Combo. In the second (regulatory) study, cats were treated with six repeated 4-week interval topical dosages of the novel formulation at 1×, 3×, or 5× multiples of the maximum recommended dose. In the first study, no significant adverse reactions were seen in the novel formulation and in the esafoxolaner groups. In the regulatory study, no significant adverse reactions were seen at 1× and 3× overdoses, and one reversible neurological adverse reaction was seen at 5× overdose. This adverse reaction was attributed to eprinomectin, because of the nature of the signs typical of avermectin toxicity [8, 23], and because of the good tolerance to a much higher dose of esafoxolaner in the first study. The results of these margin of safety studies support safe use of repeated treatments with the novel formulation and good tolerance of esafoxolaner and eprinomectin at accumulation factors of 3.2 and 2.1, respectively, which leaves long-term exposure well below the 5× maximum exposure dose.

NexGard^®^ Combo offers a convenient and safe solution to veterinarians and pet owners for the treatment of multiparasitism in cats. Unlike the simultaneous use of narrow-spectrum parasiticide products, the use of a broad-spectrum endectoparasiticide product such as this novel formulation offers precise characterization and confirmations of safety and efficacy.

## Conflict of interest

The work reported herein was funded by Boehringer-Ingelheim. The authors are current employees of Boehringer-Ingelheim Animal Heath. Other than that, the authors declare no conflict of interest. This document is provided for scientific purposes only. Any reference to a brand or trademark herein is for information purposes only and is not intended for any commercial purposes or to dilute the rights of the respective owners of the brand(s) or trademark(s).

NexGard^®^ is a registered trademark of the Boehringer-Ingelheim Group.
